# Placebo-Controlled Trial of Daily Oral Cannabidiol as Adjunctive Treatment for Cats with Chronic Gingivostomatitis

**DOI:** 10.3390/ani13172716

**Published:** 2023-08-26

**Authors:** Joana Chambel Coelho, Noélia Duarte, Andreia Bento da Silva, Maria do Rosário Bronze, Lisa Alexandra Mestrinho

**Affiliations:** 1 Faculdade de Medicina Veterinária, Universidade de Lisboa, Avenida da Universidade Técnica, 1300-477 Lisbon, Portugal; lisamestrinho@gmail.com; 2 iMed.ULisboa—Research Institute for Medicines, Faculdade de Farmácia, Universidade de Lisboa, Avenida Prof. Gama Pinto, 1649-003 Lisbon, Portugal; mduarte@ff.ulisboa.pt (N.D.); abentosilva@ff.ulisboa.pt (A.B.d.S.); 3 DCFM—Departamento de Ciências Farmacêuticas e do Medicamento, FFULisboa, Faculdade de Farmácia da Universidade de Lisboa, Avenida Prof. Gama Pinto, 1649-003 Lisbon, Portugal; mrbronze@ff.ulisboa.pt; 4 IBET—Instituto de Biologia Experimental e Tecnológica, Avenida da República, Quinta-do-Marquês, Estação Agronómica Nacional, Apartado 12, 2780-157 Oeiras, Portugal; 5 CIISA—Centro de Investigação Interdisciplinar em Sanidade Animal, Faculdade de Medicina Veterinária, Universidade de Lisboa, Avenida da Universidade Técnica, 1300-477 Lisbon, Portugal; 6 All4AnimalS—Laboratório Associado para a Ciência Animal e Veterinária, 1300-477 Lisboa, Portugal

**Keywords:** feline chronic gingivostomatitis, cannabidiol, cat, pain, dental extractions

## Abstract

**Simple Summary:**

A placebo-controlled study was conducted to evaluate the clinical efficacy and safety of a commercially available cannabidiol (CBD) oral formulation as an adjunctive treatment for pain management of feline chronic gingivostomatitis (FCGS). The results suggest that CBD, included in a multimodal approach to FCGS, was beneficial and safe since those cats medicated with CBD had a significantly higher level of comfort and activity as perceived by the owners.

**Abstract:**

A placebo-controlled study evaluated the clinical efficacy and safety of a commercially available cannabidiol (CBD) oral formulation as an adjunctive treatment for pain management for feline chronic gingivostomatitis (FCGS). CBD was included in a multimodal treatment routinely performed on client-owned cats with FCGS that were submitted to dental extractions. Twenty-two cats were consecutively included in the study. The first group was treated using a fixed dosage of 4 mg per cat every 12 h for 15 consecutive days, and the second received a placebo of similar features. Treatments began 2 h before dental extractions. Pain and disease severity were assessed at days 0 and 15 using the Composite Oral Pain Scale (COPS-C/F) and the Stomatitis Disease Activity Index score (SDAI). Weight, vital and biochemistry parameters, and analgesic reinforcement needs were also registered at the same time points. In the treated cats, blood was collected after 4, 8, and 12 h to determine CBD serum concentrations using ultra-high-performance liquid chromatography–mass spectrometry (UHPLC-MS/MS). After data analysis using mixed models, a significant improvement in the SDAI scores of cats medicated with CBD was found. The protocol is safe since severe adverse effects and biochemical changes were not observed during the treatment period. This study suggests that the cats benefited from this treatment.

## 1. Introduction

Feline chronic gingivostomatitis (FCGS) is a highly painful and debilitating oral inflammatory disease associated with chronic pain. Affected cats usually have moderate to severe pain and may show, among other clinical signs, ptyalism, halitosis, weight loss, irritability, and decreased activity [1,2]. As the disease is considered to have a multifactorial etiology, its definitive treatment and/or clinical control remain a challenge. Although dental extractions are associated with a significant remission of clinical signs, most cases still need chronic management of inflammation and pain [3].

Cannabidiol (CBD) is the most well-known non-psychoactive phytocannabinoid, with concentrations in cannabis plants ranging from 0.3% to 4.2%. It is a substance with several increasingly recognized therapeutic benefits and may present anticonvulsant, anti-inflammatory, analgesic, and anxiolytic effects, among others [4]. CBD pain and inflammatory modulation occur through the activation of the CB1, CB2, TPRV1, and glycine receptors of the endocannabinoid system [5,6,7,8]. CBD-based products have been successfully used on dogs and cats, particularly for the treatment of chronic and cancer-related pain, and research on its pharmacokinetics and safety has been increasingly reported [9,10]. However, knowledge about the efficacy of CBD in cats with chronic or inflammatory diseases is still scarce.

This study aimed to evaluate the safety and efficacy of a commercially available CBD-based oral powder formulation for 15 days of sustained treatment. The research question was as follows: is a CBD formulation beneficial and safe for continuous use in the postoperative period in cats with chronic gingivostomatitis?

## 2. Materials and Methods

### 2.1. Animals, Treatment Protocol, and Blood Sampling

The inclusion criterion was diagnosis of FCGS. The diagnoses were clinical, based on the medical history, clinical presentation, and existence of histopathology to discard differentials. On the observation of the oral cavity, in addition to gingivitis, these cats had inflammation that extended beyond the mucogingival junction across the palatoglossal folds and oropharynx. Exclusion criteria included the presence of non-associated systemic comorbidities that could lead to preoperative hematological and biochemical parameter deviations, such as renal, gastroenteric, hepatic, and neoplastic disease. All animals medicated with immunosuppressant drugs (glucocorticoids or cyclosporine) or recently vaccinated were also excluded, as well as cats with aggressive behavior. All cases were sequentially included in the study and sequentially distributed among groups as well, with first cases and second controls. Diversion from treatment for any reason resulted in exclusion from the study. The owners were not aware if their animal was receiving CBD formulation or a placebo and therefore signed an informed consent with all the information about the study design and goal. The Institution’s Ethics and Animal Welfare Committee of the Faculty of Veterinary Medicine of the University of Lisbon approved the trial.

The cats were divided into two groups, the CBD group, which was treated with the CBD powder formulation (Anibidiol Plus, Virbac, Carros, France, 8 mg of CBD in 5 g of powder), and the placebo group, which was given a placebo of similar features and presentation. Both formulations were given in individual blank tubes containing 2.5 g of powder per dose.

CBD or the placebo formulation was administered for 15 days using a fixed dose of 4 mg of CBD per cat every 12 h. Treatment began 2 h before surgery with the oral administration of CBD or placebo (diluted in 2 mL of water). All animals were fasted for 6 to 8 h prior to the first administration and were not fed until 6 h after initial dosing. All cats were pre-medicated for surgery with methadone (0.5 mg/Kg IM), ketamine (5 mg/Kg IM), and dexmedetomidine (0.05 mg/kg IM) and induced with propofol, maintaining anesthesia with a mixture of isoflurane and 100% oxygen. Before starting the dental extractions, nerve blocks were performed with lidocaine (1 mg/Kg), antibiotic cefazolin (22 mg/Kg EV), and nonsteroidal anti-inflammatory meloxicam (0.2 mg/kg SC) administered. For post-surgical recovery, all animals were hospitalized for a period of 24 h. Post-surgical medications prescribed included an antibiotic (clindamycin 11 mg/kg PO or cefovexin 8 mg/kg SC) for 14 days, an analgesic (buprenorphine 0.02 mg/kg PO) for 3 days, nonsteroidal anti-inflammatory medication (meloxicam 0.05 mg/kg PO) for 5 days, and a local antiseptic gel (chlorhexidine 0.07%). In case there was a need for additional pain control, during the 15 days of treatment, buprenorphine (0.02 mg/kg) was given orally and registered as an analgesic rescue in both groups.

At the beginning of the study, we collected 2 mL of blood to determine biochemical parameters, namely, albumin, alanine aminotransferase (ALT), aspartate aminotransferase (AST), gamma-glutamyl transferase (GGT), creatinine, and urea. These analyses were part of the inclusion criteria and repeated at the end of the trial.

Three 1 mL blood samples were also obtained to determine CBD serum concentration at three time points, 4, 8, and 12 h, using UHPLC (ultra-performance liquid chromatography coupled to mass spectrometry) using CBD-d3 as the internal standard. Samples were placed into a gel clotting tube, rested for 20 min, and centrifuged for 10 min at 2000× *g* to obtain the serum. The serum was stored in Eppendorf tubes at −80 °C until analysis at the end of the study period. CBD oral formulation was also analyzed using the same techniques. Technique details are provided in Supplementary File S1.

Clinical examinations were performed in both groups prior to the surgery and after 15 days. The following physiological parameters were registered: cardiac and respiratory rates and arterial pressure and weight. The Stomatitis Disease Activity Index (SDAI) [2,11,12] (Supplementary File S2) and the Composite Oral Pain Scale (COPS-C/F) [13] (Supplementary File S3) were collected at both time points. The SDAI score is formed by two questionnaires: one for the tutor, which assesses appetite, activity, grooming, and comfort, and the other for the clinician assessing the severity of inflammatory lesions. It establishes a numerical evaluation of the disease’s state to observe progression over time in response to any treatment strategies chosen [2,11,12]. Like the SDAI, the COPS-C/F also has two different scores, the owner’s COPS-C/F, a 25-point scale that includes the evaluation of changes in feeding behavior, interaction, grooming, activity/mobility, and specific behaviors, and a clinician’s inquiry (28-point scale) [13]. The scores were registered by 2 observers (J.C.C. and L.A.M.). Only one final measurement, which was blinded to the study, was defined by L.A.M.

### 2.2. Statistical Analysis

Sample size calculation was performed based on a power analysis, 80% power, alpha and beta of 0.05, 95% confidence interval for a score change of 6 out of 30 points, and a standard deviation of 3 [14]. The minimum number of individuals determined per group was 8.

A commercially available software package (Microsoft Excel, version 16.49 for Mac) was used to register data and perform all descriptive analyses. The open-resource statistical program R Commander for Mac version 4.1.2 was used for inferential statistics. Generalized linear mixed models (GLMMs) were performed for all variables except to compare the number of analgesic rescues and the percentual response rate between the two time points (percentual response rate = ((initial value − final value)/final value) × 100), where a *t*-test was used. To evaluate the possible relationship between the serum concentration of CBD with treatment response, we used a Spearman correlation test. The Spearman’s treatment response was determined as the difference of scores between the two time points. For the GLMMs, the animal was used as the random category, and the placebo group and the time on the first evaluation were used as the reference categories. A *p*-value < 0.05 was considered significant for a 95% confidence interval. Pharmacokinetic analysis was performed using the Mac version 9 of the statistical package GraphPad Prism.

## 3. Results

Twenty-two cats were included in the study (ten females and twelve males), with an average age of 5.7 years (range: 1–13 years) and an average weight at the beginning of the study of 4.188 kg (range: 2.420–5.790 kg). None of the animals were positive for feline leukemia virus (FeLV), and three cats were positive for feline immunodeficiency virus (FIV) (one in the CBD group and two in the placebo group).

Depending on the clinical presentation, partial or total dental extractions were performed. An average of 19 dental extractions (minimum 12 and maximum 29) were performed in the CBD group and 22 (minimum 13 and maximum 30) were performed in the placebo group.

**Figure 1:**
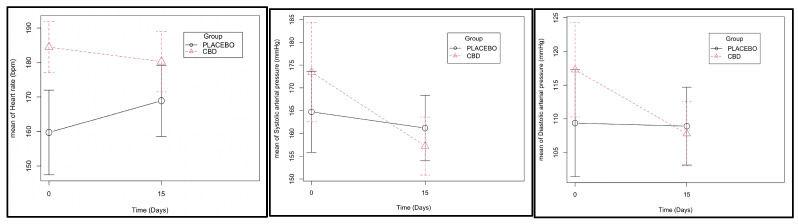
Cardiac rate and systolic and diastolic blood pressure measurements at day 0 and day 15 of CBD administration (effect of the group–time interaction with *p* values of 0.081, 0.293, and 0.287, respectively (Supplementary Table S1)).

**Figure 2:**
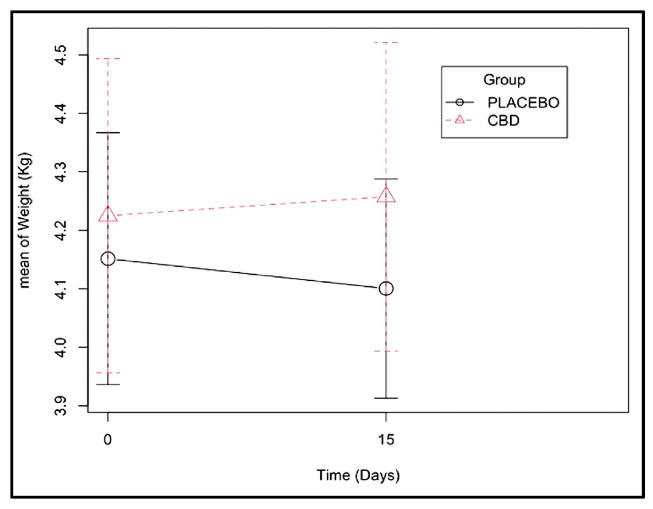
Weight change observed from days 0 and 15 of CBD administration (effect of the group–time interaction with a *p*-value of 0.121 (Supplementary Table S2)).

Fifteen days after dental extractions, both heart rate and arterial pressure levels seemed to be inferior in the CBD group (Figure 1 and Supplementary Table S1), and a more abrupt weight loss was observed in the placebo group (Figure 2 and Supplementary Table S2). However, these differences were not significant.

At the beginning of the treatment, individuals from the CBD group had lower (*p* = 0.027) AST values than the placebo group, resulting in a group effect. No differences were found in the other biochemical parameters (albumin, ALT, GGT, creatinine, and urea). In the CBD group, all biochemical parameters did not change significantly between days 0 and 15, but albumin values tended to increase in the CBD group and decreased in the placebo group (Supplementary Table S3).

After the administration of CBD, no adverse effects were observed in the cats under the care of their owners, although significant salivation, licking, and head shaking were observed by the clinician, at the time of CBD administration, in five cats. Diarrhea was observed by the owner of one cat, while vomiting food and hairballs was observed in two other cats. No other anomalies were noted on the physical examinations throughout the study or reported in the owners’ inquiries about other relevant changes, including behavior.

**Figure 3:**
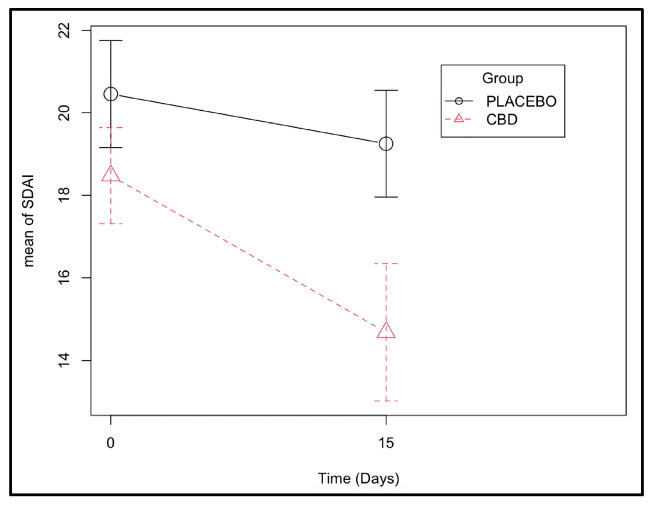
Stomatitis Disease Activity Index scores determined at days 0 and 15 in the CBD and placebo groups (effect of the group–time interaction with *p*-value < 0.001 (Supplementary Table S4)).

During the observation period, the effect of the CBD administration on recovery over time was more significant than the effect of time alone, as shown by the improvement in SDAI scores (*p* < 0.001 for the interaction group:time and *p* = 0.003 for the effect of time alone). Looking at the coefficients, it was possible to verify that on average, the CBD group had 2.6 points less in the SDAI score than the placebo group at the end of the 15-day treatment (Figure 3 and Supplementary Table S4).

**Figure 4:**
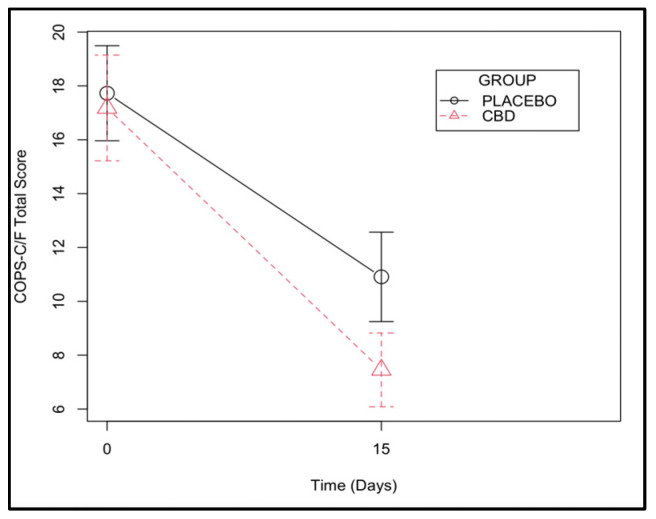
Composite Oral Pain Scale for cats and dogs (COPS-C/F) assessed at days 0 and 15 in a group of cats with chronic gingivostomatitis.

The effect of time on pain levels was significant (*p* < 0.001), with both groups having a marked relief of oral pain after the surgery using the COPS-C/F. The CBD group scored an average of 3 points less on the scale compared with the placebo group, although the difference was not statistically significant (*p* = 0.090) (Figure 4 and Supplementary Tables S5 and S6).

**Figure 5:**
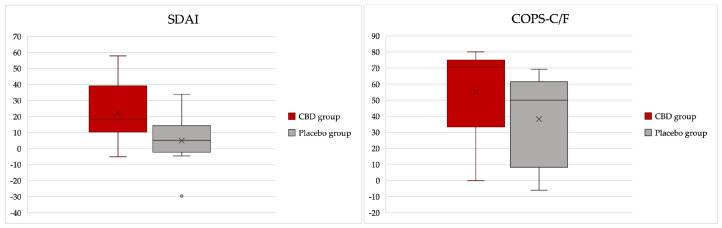
Box plots illustrating the percentual response rates on Stomatitis Disease Index (SDAI) and Composite Oral Pain Scale for cats and dogs (COPS-C/F) assessed at days 0 and 15 in a group of cats with chronic gingivostomatitis.

The average percentual response rate in the SDAI scores for the CBD group was 22.5% (standard deviation (s.d.) of 18.7), and for the placebo group, it was 4.9% (s.d. of 15.9) and significantly different (*p* = 0.028) (Figure 5). Indeed, one case in the CBD group increased on the SDAI index at day 15, as did five cases in the placebo group. The average percentual response rate on the COPS-C/F for the CBD group was 55.2% (s.d. of 27.06), and for the placebo group, it was 38.2% (s.d. of 28.8) but not significantly different (*p* = 0.169) (Figure 5). None of the cats increased their COPS-C/F scores in the CBD group, but one case did in the placebo group (Supplementary Table S6).

The average amount of rescue analgesia registered throughout all time points was 1.1 and 1.3 in the CBD and placebo groups, respectively. No significant differences were observed (*p* = 0.204).

CBD serum concentrations were variable among cats 4, 8, and 12 h after CBD administration (from 0.11 to 12.86, 0.23 to 34.81, and 0.22 to 10.00 ng mL^−1^, respectively (Supplementary Table S7)). There was a weak relationship between the dose administered and serum concentration (Spearman’s rho (rs) = 0.10023, *p* = 0.769) and a weak association between the serum concentration (ng/mL) and the treatment response assessed using the SDAI (rs = 0.20909, *p* = 0.537) scores. The relationship between serum concentration and the COPS-C/F was negligible (rs = 0.0977, *p* = 0.77505).

## 4. Discussion

The interest in CBD’s therapeutic applicability for pets has been increasing, and therefore the knowledge about its pharmacokinetics and clinical efficacy is of utmost importance. Although there are previous reports about the pharmacokinetics of CBD in cats, there is still a need for further research, especially regarding the kinetics, safety, and efficacy of different formulations, routes, and long-term treatments, among other things [10]. The formulation used in this study is not a medical product approved by the Food and Drug Administration (FDA) nor by European Medicines Agency (EMA). It was a product containing CBD that was approved as a complementary feed for companion animals at the time of the study. As defined by law, tetrahydrocannabinol (THC) concentrations were below 0.3%. In the study, the CBD content was determined by the authors using HPLC-MS/MS.

Dental extractions are considered the first line of treatment for FCGS since they usually lead to more sustained remission of clinical signs. However, long-term control of inflammation and pain is still needed in most cases [3]. In this context, CBD is a potentially interesting substance to be included in the multimodal approach to this disease, especially for those cats that need long-term support. Therefore, the goals of this study included the evaluation of CBD’s analgesic and anti-inflammatory efficacy as a therapeutic adjuvant in the treatment of cats with FCGS that were submitted to dental extractions. Furthermore, due to the need to provide sustained treatment for these cats, the study also aimed to evaluate the safety of a CBD formulation that was administered for 15 days. To achieve these goals, a single-blind, placebo-controlled pragmatic clinical trial was designed under the normal conditions of clinical practice with client-owned cats. For this reason, the authors made a choice to perform the therapy with a formulation already available on the market and administered in a fixed dose.

As FCGS is considered multifactorial in origin, both young and old cats can be affected, and some may carry retroviral disease and/or other viral infections such as feline calicivirus (FCV) and feline herpesvirus (FHV-1) [2,3,11,12,15,16]. As expected, both groups included mostly adult cats living in a multi-cat environment and/or originated from shelters, as previously described by others [17]. Three cats were of younger ages, with a history of juvenile gingivitis and periodontitis that progressed to FCGS. This association also has been previously reported [18,19].

All cats were tested for FIV and FeLV viruses due to their potential systemic impact beyond FCGS. No FeLV-positive cat was included in the study since co-infection with this virus is associated with a different response rate to dental extraction [16]. On the contrary, FIV or FCV infection does not seem to be related to a different response rate to dental extractions compared with those not infected [16,17]. Regardless, three animals were positive for the FIV virus—one in the CBD group and two in the placebo group—and FCV was diagnosed in one cat of the CBD group.

The results obtained in this study support the hypothesis that cats with FCGS benefit from oral administration of CBD and that this substance might be considered in the multimodal treatment approach to this disease. The main observations relate to the significant percentual improvement of the SDAI scores obtained at day 15 for the CBD group, and the significant group:time interaction obtained in the SDAI GLMM analysis. Other observations were merely suggestive but will be discussed below since they can also be related to CBD.

As expected, both groups improved their SDAI and COPS-C/F scores at the end of the study due to the surgical treatment itself. However, the average percentual response rate on the SDAI index of the CBD group was significantly superior to the placebo group. In addition, these findings were reinforced in the GLMM analysis, highlighting the adjuvant analgesic and anti-inflammatory benefits of this substance. The SDAI index includes a 3-point maximum score attributed to the owner’s perception of comfort, a 3-point maximum score attributed to weight balance, and a 24-point maximum score for the severity of inflammatory lesions, meaning that the SDAI is more than a pain scale, evaluating the overall inflammation and reinforcing the benefit of CBD for the control of inflammation. On the other hand, the COPS-C/F is truly a pain scale. In this assessment, the COPS C/F scores were not significantly attributed to CBD alone and instead were attributed more to the effect of time, although a tendency was observed for the treated cats to score 3 points less on average than the placebo group, and it was close to statistical significance (*p* = 0.090). The circumstances of surgery, which included the extraction of teeth, may have influenced the points obtained on the pain scale since teeth were absent at the second point of evaluation. This fact might have contributed more to the decrease in the score and indirectly contributed to the effect of time. Finally, the lower amount of rescue analgesia in the CBD group and the consistent negative weight coefficient observed in the placebo group at the end of 15 days, albeit suggestive, are worth mentioning and point in this same direction. Future studies including a higher number of subjects are needed to confirm this tendency.

CBD is a multitarget drug that interacts with diverse signaling systems that go beyond cannabinoid receptor(s) interactions. One of its most important roles includes the enhancement of the intrinsic signaling pathway of anandamide (an endocannabinoid) by decreasing its cellular re-uptake and FAAH-mediated catabolism (fatty acid amide hydrolase-mediated catabolism) [20]. It can enhance serotonergic activity through 5-HT1a (Serotonin or 5-hydroxytryptamine receptors), down-regulate cyclooxygenase enzymes, and interact with multiple receptors, channels, and transporters, such as glycine receptors, TRPA1 (transient receptor potential ankyrin 1), TRPV (transient receptor potential vanilloid) 1 and 2, GPR55 (a cannabinoid receptor), TRPM8 (transient receptor potential of melastatin type 8 channel), or ENT (equilibrative nucleoside transporter) [6]. These mechanisms lead to the anti-inflammatory, analgesic, and anxiolytic effects observed in this group of cats. Other effects have been reported in other species, including antiemetic or antiepileptic effects [4,21]. To the best of our knowledge, there are no published studies on the effectiveness of CBD for the treatment of a chronic and painful inflammatory disease like FCGS in cats.

It is worth noting that some non-significant differences observed between the groups can also be attributed to the effect of CBD. Both heart rate and arterial pressure tended to decrease in the CBD group at the end of the observational period. Indeed, for heart rate, the differences were close to statistical significance, *p* = 0.052 (Supplementary Table S1). This fact is not relevant to the pain assessment itself, but this observation is relevant since it can be attributed to CBD’s anxiolytic effects. The placebo group showed higher levels of arterial pressure and heart and respiratory rates compared with normal range values, reflecting the high levels of stress and discomfort of the cats that were perceived during consultation. Further studies are needed with an increased sample size to evaluate if CBD can reduce anxiety in cats.

CBD oral administration has been increasing for pets, raising important concerns in terms of efficacy and safety, especially for cats, where CDB’s clinical benefit is still quite unknown. In this study, no significant changes in the biochemical parameters were observed and no major adverse effects were registered, which proves the safety and tolerability of CBD at this dose regimen. It is worth mentioning that, after 15 days of treatment, the mean values of albumin in the placebo group showed a marked decrease that was not observed in the CBD group, which, on the contrary, showed a slight increase. Since albumin is a negative acute-phase protein, it is possible that these changes could be related to the anti-inflammatory effect of CBD. However, given the small sample size and treatment time, it is not possible to conclude that these variations were due to CBD administration. Further studies are needed to evaluate the effect of CBD on acute-phase protein levels in FCGS.

The formulation delivered a fixed dosage of CBD per cat every 12 h to a total of 8 mg a day. This corresponded to approximately 1 mg/kg. The choice for a fixed dosage was based on two factors: the presentation of the product (a sachet) and the lack of information on the safety of a continuous regimen. Previous reports recommended a 2 mg/kg dosage, twice daily [9]. Furthermore, escalating dosages for cats have been published, and their apparent safety has only recently been discovered [10]. Our preliminary UHPLC-MS/MS assays examined the absorption of CBD from the powder oral formulation, and CBD serum concentrations were variable among individuals and inferior to what has been previously reported [9,10]. The weak correlation found between the dose administered per kg and the serum concentration obtained can be explained by CBD’s high lipid solubility [9,10]. The powder formula could have decreased gastrointestinal absorption compared with oil. Additionally, the disease status may have influenced the pharmacokinetics of the product, namely low body weight and the amount of fatty tissue [22]. More pharmacokinetic studies are needed to better understand these issues.

The CBD-based formulation studied here also contained ingredients such as vitamins B3 and B6 and Omega 3, 6, and 9, which are described as having anti-inflammatory or healing properties [23,24]. However, previous studies failed to prove that omega 3:6 supplements provide therapeutic anti-inflammatory properties as a food supplement for cats with chronic gingivostomatitis [25]. Vitamins B3 and 6 can play a role as adjuvants in inflammation, although they do not have proven therapeutical advantages alone to the authors’ knowledge. Furthermore, the dosage of these vitamins provided in half a sachet (8 and 1.25 mg, respectively) was significantly inferior to the Association of American Feed Control Officials’ (AAFCO’s) daily recommendations (60 mg/kg and 4 mg/kg, respectively). Since the placebo supplementation did not include any of these supplements, the authors cannot completely exclude that the effects observed could be exclusively attributed to CBD alone or could be potentiated by these supplements. Further studies aiming to exclude potential bias attributed to the effects of these supplements should be carried out.

Salivation, licking, and head shaking were observed after administration of the formulation dissolved in water. These reactions could be associated with the taste of the powder formulation since it was not verified when the product was dissolved in food. Other observed effects included diarrhea, vomiting, and increased production of hairballs, which have also been previously described [9,10]. In this study, it was not possible to conclusively relate these effects to CBD, since other medications were also given in the immediate post-operatory period, namely one anti-inflammatory (5 days), one antibiotic (14 days), and another analgesic (3 days). Potential side effects of CBD that have been reported in cats are limited, being mostly gastrointestinal, such as diarrhea and vomiting, which were also observed here [10]. However, other effects include sedation and possible interactions with other medications. Sedation was not observed in this case series, nor were any interactions with medications. However, a possible interaction could be anticipated with the opioid methadone, since it is metabolized in the liver by the same group of cytochromes P450 enzymes that is known in humans’ CYP3A4 and CYP2B6. This interaction can increase the bioavailability of methadone, increase sedative effects, and potentially impact CBD metabolism. The authors do not know of any research aiming at studying this effect [26]. It is worth mentioning that most of the side effects observed in cannabinoid formulations are secondary to the THC level, and it is mandatory to assure that commercially available formulations have THC levels below 0.3%, as defined by law. Further investigation and regulation work must be conducted to provide a medical formulation, and not food supplements that carry only the CBD active principle, to the market.

The limitations of the study must also be discussed. Although this study was not randomized and had a relatively small sample size, one clinician and the owners were blinded to the administration of the drug, which counterbalances a possible observation bias. It would be best to have two clinicians blinded to the study. The SDAI index and COPS C/F scale are partially validated assessment tools. However, the SDAI is the clinical assessment tool mostly used in FCGS studies [2,27,28,29,30,31], and the COPS C/F is the only pain score developed to evaluate oral pain [13]. Variability between the groups resulted from the small sample size, among other factors. The baseline characteristics were not the same between the groups. The use of GLMM analysis and the assessment of the percentual balance contributed to overcoming the variability within the groups and the possible interactions. The choice of a fixed dosage and possible time variations between administration (12 h +/− 2 h) also increased variability among individuals and impacted its effectiveness. The authors attempted to analyze if there were any associations between the systemic concentration of CBD and the clinical improvement (SDAI and COPS-C/F points) and compare treated individuals. The analysis performed failed to prove a positive relationship between an increased serum concentration and clinical improvement. Only a weak positive association was observed between the CBD concentration and SDAI improvement, but it was not observed for the COPS-C/F. Finally, the caregiver placebo effect must also be recognized.

## 5. Conclusions

Oral administration of cannabidiol seems to benefit the post-operatory recovery of cats with FCGS as part of a multimodal approach to this disease. The cats significantly improved in their levels of comfort and inflammation. It was also found that the administration of CBD at this dosage for 15 days did not cause a systemic impact or significant adverse effects.

This clinical trial reinforces the need to continue clinical and pharmacological research on the use of CBD for cats for inflammatory diseases with chronic pain, such as FCGS. Even though these short-term results are encouraging, further studies with larger groups and higher dosages are needed to identify the long-term effects of CBD treatment.

## Data Availability

All raw data involved in this study can be provided by contacting the corresponding author of the article.

## Supplementary Materials

The following supporting information can be downloaded at https://www.mdpi.com/article/10.3390/ani13172716/s1: Supplementary Table S1: Statistical analysis of the physiological parameters; Supplementary Table S2: Statistical analysis of the weight; Supplementary Table S3: Statistical analysis of the blood biochemistry; Supplementary Table S4: Statistical analysis of the SDAI; Supplementary Table S5: Statistical analysis of the COPS-C/F; Supplementary Table S6: SDAI and COPS-C/F scores obtained for every individual of each group and the treatment response rate at day 15; Supplementary Table S7: CBD descriptive values for oral administration per kg and serum concentrations for all individuals at the different assessment time points (at 4, 8, and 12 h after administration); Supplementary File S1: CBD serum quantitation using UHPLC-MS/MS; Supplementary File S2: Stomatitis Disease Activity Index (SDAI) scores; Supplementary File S3: Composite Oral Pain Scale-Canine/Feline (COPS-C/F).
