# Supplementary material for: Placebo-Controlled Trial of Daily Oral Cannabidiol as Adjunctive Treatment for Cats with Chronic Gingivostomatitis
Source: Animals (Basel). 2023 Aug 26;13(17):2716. doi: 10.3390/ani13172716 (PMC10487179; doi:10.3390/ani13172716)
Supplement: Supplementary file 1 [file animals-13-02716-s001.zip › Supplementary file S1.pdf]

Supplementary file S1 - CBD serum quantitation by UHPLC-MS/MS

Chromatographic and mass spectrometry conditions

The UHPLC-MS/MS analyses were performed on a Waters Acquity™ (Waters®, Ireland) comprising a binary pump, an on-line solvent degasser, an autosampler, and a column oven. Chromatographic separation of the target compound was performed using a reverse phase column (Purospher® STAR RP-18 2 µm, 2.1 x 50 mm) at 40 °C. The mobile phase consisted of water with 0.1 % formic acid (A) and acetonitrile (B) at a constant flow rate of 0.3 mL min^{-1}, with the following elution program: 0 – 2 min at 50% B; 2 – 5 min from 50 to 95% B; 5 – 7 min at 95% B; 7.1 – 10 min at 50% B. The injection volume was 10 µL. Mass spectrometry detection was carried out on a Waters Acquity™ (Waters®, Ireland) triple quadrupole using an electrospray ionization source operating in positive mode at 120 °C, and applying a capillary voltage of 3.0 kV. High-purity nitrogen (N_2) was used both as drying gas and as a nebulizing gas. Ultra-high purity Argon (Ar) was used as the collision gas. The samples were analyzed in MRM (multiple reaction monitoring) mode to improve selectivity and sensitivity. The MS/MS conditions were optimized to maximize the transition signals for CBD and CBD-d3, used as internal standard. Two transitions were used to quantitate (MRM1, 315 > 193 for CBD and 318 > 196 for CBD-d3) and confirm the identification (MRM2, 315 > 135 for CBD and 318 > 135 for CBD-d3), with a maximum deviation of 30% between the MRM1/MRM2 ratio (ion ratio). MassLynx® 4.1 software was used for data acquisition and processing.

Preparation and quantitation of CBD in the formulation

CBD was quantitated in two different granulated sachets of Anibidiol plus, Virbac. The granules were crushed into powder and 6-12 mg were rigorously measured and dissolved in methanol in order to obtain a theoretical CBD concentration of 10 µg mL^{-1}. After being taken to an ultrasonic bath for 10 minutes, the solution was diluted 1:100 in acetonitrile (final granulate concentration of 6.25 mg mL^{-1} and theoretical CBD concentration of 100 ng mL^{-1}) and filtered by 0.20 µm PTFE filters before analysis. CBD was quantitated by comparing the areas with three independent CBD solutions at 100 ng mL^{-1} in acetonitrile. The concentration of CBD in the granules was 1.57 ± 0.02 mg g-1, corresponding to the theoretical concentration of 8 mg of CBD in the 5 g of powder per sachet.

Sample preparation

CBD was extracted from serum following a method previously described [9], with a few adaptations. Briefly, 100 µL of sample was transferred to 2 mL glass vials and 250 µL of an icy solution (-20 °C) of CBD-d3 at 4 ng mL^{-1} in acetonitrile and 200 µL of water was added. The solutions were later homogenized in the vortex, before (15 seconds) and after (1 minute) adding 1 mL of hexane. After phase separation at 4 °C, 800 µL of the organic phase (upper layer) were removed for glass vials. The extracts were evaporated until dryness, reconstituted in 50 µL of acetonitrile, filtered by PTFE 0.2 µm syringe filters, and injected into the equipment. Each sample was extracted in duplicate.

Method validation

Four calibration curves were prepared in acetonitrile using a CBD commercial standard, considering two different concentration ranges, namely from 0.2 to 2 ng mL^{-1} ($y = 156.60x - 9.79$), and from 2 to 50 ng mL^{-1} ($y = 154.50x + 32.22$). Each curve was analyzed in the equipment at least in duplicate.

In order to determine the limit of detection (LOD) of CBD in serum, six independent stock solutions of CBD were diluted in a blank matrix (extracted serum samples from healthy control cats), until a signal-to-noise ratio of 3:1. The limit of quantitation (LOQ) was defined as the lowest concentration of CBD to give acceptable (< 20%) precision and accuracy errors. To verify

the method's precision and accuracy, standard CBD solutions were prepared at 0.2, 2, and 20 ng mL^{-1} and analyzed among every 20 injections of samples.
The influence of the matrix on the signal of the equipment (matrix effect) was also evaluated, comparing the signals of CBD solutions prepared in solvent and two blank matrixes, at 0.2, 2, and 20 ng mL^{-1}.
In order to determine the recovery of the extraction procedure, six serum samples (100 μL) from healthy control cats were fortified with 10 μL of CBD standard solutions at 2, 20, or 200 ng mL^{-1}, and extracted as previously described. Therefore, after evaporation and reconstitution of the samples in 50 μL of acetonitrile, the theoretical concentrations of CBD were 0.4, 4, and 40 ng mL^{-1}, respectively.
