# Supplementary material for: Placebo-Controlled Trial of Daily Oral Cannabidiol as Adjunctive Treatment for Cats with Chronic Gingivostomatitis
Source: Animals (Basel). 2023 Aug 26;13(17):2716. doi: 10.3390/ani13172716 (PMC10487179; doi:10.3390/ani13172716)
Supplement: Supplementary file 1 [file animals-13-02716-s001.zip › Supplementary file S2.pdf]

Stomatitis Disease Activity Index (SDAI) Score

Owner Evaluation

**Appetite**	3 = eats only pureed food, or only when hand fed<br>2 = eats wet food; cannot eat dry food<br>1 = eating wet and dry food, but less than normal amount<br>0 = eating normally
**Activity Level**	3 = no interest in people or other pets, spends most of time sleeping<br>2 = low activity level, but will play occasionally when engaged by people or other pets<br>1 = plays spontaneously, but not frequently<br>0 = normal activity level (playful and active)
**Grooming Behavior**	3 = will not groom<br>2 = grooms occasionally but not at 'pre-illness' level<br>1 = grooming excessively<br>0 = grooming normally
**Perceived Comfort**	On a scale of 0-3, with 0 being most comfortable and 3 being most painful, rank your cat's present comfort level: _____

Scoring Criteria for Stomatitis Disease Activity Index (SDAI) Tabulation.

**Owner Evaluation***	The average of the scores above
**Weight (compared with most recent visit)**	0 = gain > 0.5kg<br>1 = gain > 0.25kg but < 0.5kg<br>2 = < 0.25 kg gain<br>3 = weight loss
**Inflammation (specified sites as graded by clinician)***	0 = none<br>1 = mild<br>2 = moderate<br>3 = severe

* Intermediate value scores were accepted in these categories; As a result, the final SDAI scores may not be whole numbers

Stomatitis Disease Activity Index (SDAI) Final Score Tabulation.

**STOMATITIS DISEASE ACTIVITY INDEX**	**Day 0**	**Day 15**
Owner evaluation		
Weight (compared to most recent visit)		
Maxillary buccal mucosal inflammation		
Mandibular buccal mucosal inflammation		
Maxillary attached gingival inflammation		
Mandibular attached gingival inflammation		
Inflammation lateral to palatoglossal folds		
Molar salivary gland inflammation		
Oropharyngeal inflammation		
Lingual and/or sublingual inflammation		
**TOTAL SCORE (max = 30)**		
