# Supplementary material for: Placebo-Controlled Trial of Daily Oral Cannabidiol as Adjunctive Treatment for Cats with Chronic Gingivostomatitis
Source: Animals (Basel). 2023 Aug 26;13(17):2716. doi: 10.3390/ani13172716 (PMC10487179; doi:10.3390/ani13172716)
Supplement: Supplementary file 1 [file animals-13-02716-s001.zip › Supplementary file S3.pdf]

Composite oral pain scale-canine/feline (COPS-C/F)

Owner Specific Questionnaire

1st Evaluation	Follow-up
**What type of food do you give to your pet?**<br>Dry<br>Wet<br>Mixed	
**Q1. Considering the food that you give and compared with the past, your pet:**<br>0 Eats normally<br>1 Eats more slowly<br>2 Eats less<br>3 Has some unusual behaviors (e.g., retracts/complains while chewing; drops the bite while chewing, other)<br>4 Does not eat at all	
**Q2. Have you noticed any change in your pet's feeding behavior compared with the past?**<br>0 No change<br>1 Shows interest in food but after a few bites goes away from it<br>2 Shows less interest in food<br>3 It is uninterested in food	
**Q3. Have you noticed any change in your pet's willingness to interact/play with people or other pets compared with the past?**<br>**0 No change**<br>1 Less active than usual, but still willing to interact/play<br>2 Depressed, less willing to interact/play<br>3 Nervous, anxious, sometimes aggressive toward people/other pets<br>4 No longer interacts with people/other pets; tends to hide or to lay in its kennel	
**Q4. Have you noticed any change in your pet's personal hygiene (grooming, licking) compared with the past?**<br>0 No change<br>1 Spends less time cleaning itself<br>2 Does not clean itself (it is dirty; the fur is disheveled)	
**Q5. Have you noticed any change in your pet's physical activity/mobility (running, walking, etc.) compared with the past?**<br>0 No change<br>1 Less willing to do physical activity<br>2 Refuses to do any physical activity	
**Q6. Have you noticed the presence of one or more of the following behaviors?**<br>1 Moans/groans<br>1 Increased aggressiveness and/or nervousness<br>1 Avoids being touched around nose/mouth<br>1 Has less interest in playing involving the use of mouth (wooden sticks, toys, etc.)<br>1 Often scratches the mouth area<br>1 Has difficulties in yawning and/or opening the mouth<br>1 Produces more saliva and/or swallows more frequently<br>1 Bad breath<br>1 Grinds its teeth<br>1 Chewing without food in its mouth	

Veterinary Specific Questionnaire

1st Evaluation	Follow-up
**Q1. The animal is:**	
0 Lively, happy	
1 Quiet	
2 Indifferent to its surroundings	
3 Nervous, anxious, scared	
4 Depressed, unresponsive to stimuli	
**Q2. When manipulating the oral cavity, the animal is:**	
0 Calm, relaxed	
1 Looks around	
2 Tries to avoid manipulations	
3 Complains	
4 Growls/hisses and/or attempts to bite/scratch	
**Q3. During the visit, did you observe any of the following?**	
1 Ptyalism	
1 Nasal discharge	
1 Resistance/difficulty in opening the mouth	
1 Crackles when manipulating the temporomandibular joint	
1 Atrophy of masseter/temporalis muscles	
1 Swelling of masseter/temporalis muscles	
1 Mouth swelling or asymmetry	
1 Sensitivity and/or increase in resistance to the eye pressure	
**Q4. While examining the oral cavity, did you observe any of the following?**	
1 Accumulation of food in the oral cavity	
1 Halitosis	
1 Spontaneous/provoked gums bleeding	
1 Dental fractures	
1 Dental malformations	
1 Tooth discoloration	
1 Tooth mobility	
1 Enamel hypocalcification or hypoplasia	
1 Hyperplasia of the gums or presence of gum tumors	
1 Ulcerative lesions of the oral mucosa/tongue/palate/gums	
1 Maxillary or peri-zygomatic fistulas	
1 Gum or mucosae fistulas	
