# Supplementary material for: Placebo-Controlled Trial of Daily Oral Cannabidiol as Adjunctive Treatment for Cats with Chronic Gingivostomatitis
Source: Animals (Basel). 2023 Aug 26;13(17):2716. doi: 10.3390/ani13172716 (PMC10487179; doi:10.3390/ani13172716)
Supplement: Supplementary file 1 [file animals-13-02716-s001.zip › Supplementary tables.pdf]

Supplementary Table S1- Statistical analysis of the physiological parameters.

GLMM	Coefficient	Std. Error	t value	p value	95% CI for coefficient	
**Fixed effects**					**2.5%**	**97.5%**
			**Cardiac rate (bpm)**			
**Intercept**	159.727	8.995	17.758		142.098	177.357
**Group (CBD)**	24.727	12.725	1.943	0.052	-0.213	49.668
**Time (Day 15)**	7.182	5.418	1.695	0.090	-1.438	19.802
**Group:Time**	-13.364	7.664	-1.744	0.081	-28.384	1.657
			**Respiratory rate (rpm)**			
**Intercept**	54.909	4.506	12.186		46.077	63.741
**Group (CBD)**	-1.818	6.373	-0.285	0.775	-14.308	10.672
**Time (Day 15)**	7.273	4.961	1.466	0.143	-4.452	16.997
**Group:Time**	13.091	7.017	1.866	0.062	-0.662	26.844
			**Systolic blood pressure (mmHg)**			
**Intercept**	164.727	6.994	23.553		151.019	178.435
**Group (CBD)**	7.043	9.687	0.727	0.467	-11.944	26.029
**Time (Day 15)**	-3.545	8.287	-0.428	0.669	-19.787	12.696
**Group:Time**	-12.759	12.134	-1.052	0.293	-36.541	11.022
			**Diastolic blood pressure (mmHg)**			
**Intercept**	109.364	5.355	20.422		94.868	119.860
**Group (CBD)**	7.122	7.433	0.958	0.334	-7.445	121.690
**Time (Day 15)**	-0.455	6.025	-0.075	0.940	-12.263	11.354
**Group:Time**	-9.380	8.815	-1.064	0.287	-26.657	7.896

Supplementary Table S2– Statistical analysis of the weight**.**

GLMM	Coefficient	Std. Error	t value	p value	95% CI for coefficient	
**Fixed effects**					**2.5%**	**97.5%**
Intercept	4.151	0.308	13.419		3.548	4.754
**Group (CBD)**	0.074	0.435	0.169	0.866	-0.779	0.927
**Time (Day 15)**	-0.051	0.038	-1.341	0.180	-0.125	0.024
**Group:Time**	0.083	0.054	1.549	0.121	-0.022	0.188

Supplementary Table S3 – Statistical analysis of the blood biochemistry.

GLMM	Coefficient	Std. Error	t value	p value	95% CI for coefficient	
**Fixed effects**					**2.5%**	**97.5%**
			**Albumin**			
Intercept	3.220	0.128	25.083		2.968	3.472
**Group (CBD)**	0.046	0.186	0.247	0.805	-0.319	0.411
**Time (Day 15)**	-0.159	0.085	-1.868	0.062	-0.326	0.008
**Group:Time**	0.176	0.123	1.427	0.154	-0.067	0.418
			**ALT**			
Intercept	26.664	3.339	7.987		20.120	33.207
**Group (CBD)**	7.576	4.838	1.566	0.117	-1.906	17.058
**Time (Day 15)**	3.500	2.679	1.306	0.191	-1.751	8.751
**Group:Time**	5.170	3.882	1.332	1.183	-2.439	12.779
			**AST**			
Intercept	26.991	2.343	11.522		22.400	31.582
**Group (CBD)**	-7.531	3.395	-2.218	0.027	-14.184	-0.878
**Time (Day 15)**	-0.136	2.654	-0.051	0.959	-5.337	5.064
**Group:Time**	1.136	3.845	0.296	0.768	-6.400	8.673
			**GGT**			
Intercept	0.855	0.353	2.419		0.162	1.547
**Group (CBD)**	0.264	0.500	0.528	0.598	-0.715	1.243
**Time (Day 15)**	0.682	0.500	1.365	0.172	-0.297	1.661
**Group:Time**	-0.173	0.707	-0.244	0.807	-1.557	1.212
			**Urea**			
Intercept	55.527	4.387	12.658		46.930	64.125
**Group (CBD)**	-3.577	6.357	-0.563	0.574	-16.036	8.882
**Time (Day 15)**	1.064	3.208	0.332	0.740	-5.223	7.350
**Group:Time**	1.716	4.648	0.369	0.712	-7.393	10.826
			**Creatinine**			
Intercept	1.363	0.114	11.905		1.138	1.587
**Group (CBD)**	-0.185	0.162	-1.146	0.252	-0.503	0.132
**Time (Day 15)**	0.050	0.062	0.809	0.418	-0.071	0.171
**Group:Time**	0.086	0.087	0.988	0.323	-0.085	0.258

Supplementary Table S4 - Statistical analysis of the SDAI.

GLMM	Coefficient	Std. Error	t value	p value	95% CI for coefficient	
**Fixed effects**					**2.5%**	**97.5%**
Intercept	20.455	1.269	16.114		17.967	22.942
**Group (CBD)**	-1.978	1.795	-1.102	0.270	-5.497	1.540
**Time (Day 15)**	-1.205	0.544	-2.215	0.003	-2.270	-0.139
**Group:Time**	-2.590	0.769	-3.368	< 0.001	-4.097	-1.083

Supplementary Table S5 - Statistical analysis of the COPS-C/F.

GLMM	Coefficient	Std. Error	t value	p value	95% CI for coefficient	
Fixed effects					2.5%	97.5%
Intercept	17.727	1.492	11.886		14.804	20.650
Group (CBD)	-0.546	2.109	-0.259	0.796	-4.679	3.589
Time (Day 15)	-6.818	1.215	-5.610	< 0.001	-9.200	-4.436
Group:Time	-2.909	1.719	-1.692	0.091	-6.278	0.460

Supplementary Table S6 – SDAI and COPS-C/F score obtained for every individual of each group, and the treatment response rate at day 15.

			SDAI			
**Group**		**CBD**			**Placebo**	
**Animal**	**Day 0**	**Day 15**	**Response Rate (%)**	**Day 0**	**Day 15**	**Response Rate (%)**
**1**	24.25	21.5	11.34	20.00	19.75	1.25
**2**	14.24	6.00	57.86	15.50	10.25	33.87
**3**	16.50	9.25	43.94	21.00	18.75	10.71
**4**	12.25	10.00	18.37	11.00	14.25	(-)29.54
**5**	16.00	9.75	39.06	24.50	23.25	5.10
**6**	19.75	20.75	(-)5.06	23.00	18.75	18.48
**7**	23.25	18.00	22.58	20.00	20.25	(-)1.25
**8**	15.5	11.00	29.03	21.75	22.75	(-)4.60
**9**	19.25	17.25	10.39	22.00	22.50	(-)2.27
**10**	22.00	18.25	17.05	27.00	24.75	8.33
**11**	20.25	19.75	2.47	19.25	16.5	14.29
			**COPS-C/F**			
**Group**		**CBD**			**Placebo**	
**Animal**	**Day 0**	**Day 15**	**Response Rate (%)**	**Day 0**	**Day 15**	**Response Rate (%)**
**1**	23	17	26.09	13	5	61.54
**2**	11	3	72.72	13	4	69.23
**3**	18	6	66.67	17	6	64.71
**4**	15	3	80.00	16	16	0
**5**	15	10	33.33	17	18	(-)5.88
**6**	12	12	0	20	18	10.00
**7**	34	9	73.53	13	5	61.54
**8**	12	3	75.00	17	8	52.94
**9**	17	5	70.59	28	14	50.00
**10**	17	4	76.47	29	15	48.28
**11**	15	10	33.33	12	11	8.33

Supplementary Table S7 – CBD descriptive values for oral administration per kg and serum concentrations for all individuals at the different assessment time points (At 4, 8, and 12h after administration).

Animal	Dosage (mg/Kg)	Time (Hours)	CBD Mean concentration (ng mL^{-1})	Standard deviation
**1**	0.87	4	6.74	0.04
		8	5.52	0.44
		12	1.92	0.14
**2**	1.00	4	1.76	0.08
		8	1.15	0.04
		12	1.01	0.04
**3**	1.65	4	10.47	0.61
		8	34.81	4.01
		12	10.00	0.59
**4**	1.02	4	12.86	0.80
		8	4.47	0.37
		12	1.60	0.10
**5**	0.69	4	2.75	0.08
		8	0.57	0.06
		12	0.74	0.05
**6**	0.84	4	12.10	0.52
		8	1.47	0.10
		12	0.80	0.06
**7**	1.03	4	1.61	0.07
		8	1.20	0.12
		12	2.40	0.16
**8**	1.18	4	1.20	0.03
		8	0.23	0.01
		12	0.22	0.02
**9**	1.00	4	4.28	0.02
		8	2.33	0.26
		12	3.47	0.16
**10**	0.83	4	6.69	0.08
		8	4.49	0.16
		12	0.96	0.00
**11**	0.82	4	0.11	0.01
		8	0.35	0.03
		12	0.50	0.00
